# Effects of diagnostic labels on perceptions of marginal cases of mental ill-health

**DOI:** 10.1371/journal.pmen.0000096

**Published:** 2024-08-28

**Authors:** Brooke Altmann, Kylo Fleischer, Jesse Tse, Nick Haslam

**Affiliations:** Melbourne School of Psychological Sciences, University of Melbourne, Parkville, Australia; University of Saskatchewan, CANADA

## Abstract

Two experimental studies (*N*s = 261, 684) investigated how diagnostic labels affect perceptions of people experiencing marginal levels of mental ill-health. These effects offer insight into the consequences of diagnostic “concept creep”, in which concepts of mental illness broaden to include less severe phenomena. The studies found consistent evidence that diagnostic labeling increases the perception that people experiencing marginal problems require professional treatment, and some evidence that it increases empathy towards them and support for affording them special allowances at work, school, and home. The studies also indicated that labels may reduce the control people are perceived to have over their problems and their likelihood of recovering from them. These findings point to the potential mixed blessings of broad diagnostic concepts and the cultural trends responsible for them. Expansive concepts may promote help-seeking, empathy, and support, but also undermine perceived agency and expectations that problems can be overcome.

## Introduction

Mental illness occupies an increasingly prominent place in our cultures. Coverage of mental health problems saturates both traditional and social media. Demand for mental health care has surged and the prevalence of some conditions has risen. In the midst of these changes, the public also appears to be better informed about mental illness and somewhat more accepting of it: Levels of mental health literacy have generally risen in recent decades [1] and levels of stigma towards some conditions have fallen [2,3].

Critics have argued that this rise in awareness and cultural attention has expanded our concepts of mental ill health. Some point to changes in official psychiatric classifications, contending that diagnostic inflation has led everyday anxiety and sadness to be pathologized as disorders [4,5] and that *DSM-5* colonized large swathes of normality [6]. Others propose that social media [7], mental health awareness campaigns [8], and broad cultural shifts [9], rather than diagnostic systems themselves, are responsible for increasingly expansive concepts of mental illness. They have raised concerns that social media platforms such as TikTok and the rising popularity of diagnostic concepts may be encouraging excessive self-diagnosis, with adverse implications such as over-use of clinical services [10].

Changes in the expansiveness of concepts of mental illness can be understood as examples of “concept creep”. Haslam [11] proposed that harm-related concepts, including mental illness, have progressively broadened their meanings in recent decades so that they now refer to a substantially wider range of phenomena than they did in earlier times. They have broadened “horizontally” to encompass a greater variety of harms and “vertically” to include less severe forms. Research using historical datasets has found substantial evidence for these semantic shifts in the domain of mental ill health. Concepts associated with mental illness, such as trauma, have broadened horizontally [9] and vertically [12], and concepts referring to negative emotional states such as anxiety and grief have increasingly been pathologized [13]. The findings lend credence to the view that diagnostic concepts have been expanding.

The concept creep of mental illness implies that diagnoses are being made more freely now than before, as the threshold for identifying problems as illnesses has fallen. Recent research supports this point, finding that people holding more expansive concepts of mental disorder were more likely to self-diagnose, even after statistically controlling for their levels of distress, impairment, mental health literacy, and stigmatizing attitudes [14]. The implications of this expanding tendency to diagnose or self-diagnose mental illness are as yet unknown. To find out, we must understand the consequences of seeing marginal levels of distress as diagnosable disorders. Examining the effects of diagnostic labeling in this marginal context is one way to do this.

The theory of concept creep suggests that broadened concepts of harm tend to have mixed blessings [11]. On the one hand, they promote care and moral concern for the person who is harmed, but on the other, they may foster fragility and victim-based identities. We predicted that similar mixed blessings might result from expanding concepts of mental illness, reflected in increased diagnostic labeling of less severe distress. Assigning a diagnostic label to a person experiencing marginal levels of mental ill-health might bring sympathy, support, and access to professional treatment, but it might also lead to their problems being seen as lasting, beyond their control, and self-defining.

Studies of diagnostic labeling provide evidence germane to these predictions. With regard to the predicted benefits, some research indicates that labeling decreases stigma, increases the humanity ascribed to people experiencing symptoms [15], and can increase lenient treatment from others as well as access to community and professional supports [16]. However, a recent review finds that labeling can either increase or decrease stigma depending on the disorder, mitigating stigma for autism but exacerbating it for schizophrenia, for example [17]. With regard to predicted costs, research indicates that using noun labels for social categories increases their perceived temporary stability [18], which in the context of diagnostic categories implies that labeling may increase the perceived persistence of people’s difficulties or decrease the perceived likelihood of recovery. This possibility is supported by research, which indicates that people who see their mental health problems as a continuing aspect of their identity are less likely to recover [19]. The possibility that diagnostic labeling undermines perceived control of people’s difficulties is supported by Ahuvia and colleagues [20], who found that self-labeling with depression by college students was associated with lower perceived control over their symptoms, controlling for the severity of those symptoms.

Vignette designs are a popular method for investigating diagnostic labeling. These designs employ brief descriptions of individuals experiencing a set of symptoms and are commonly used to assess whether people can identify the correct diagnosis. They can also be used to evaluate the effects of labeling by experimentally manipulating whether or not a label accompanies the vignette. Experimental studies of this sort have primarily focused on the effects of labels on stigmatizing responses, yielding inconsistent findings. Reviewers of the literature have argued that this work has over-relied on a few conditions–attention deficit hyperactivity disorder, schizophrenia spectrum disorders, and autism spectrum disorder–and on convenience samples [17].

The present research employed a vignette methodology to examine the potentially mixed effects of diagnostic labeling in the context of concept creep and concept breadth. It departed from previous vignette-based labeling research in several respects. First, given its focus on the expanding use of diagnostic labels, it employed vignettes that deliberately described relatively mild or marginal instances of mental disorders. In these cases, it is ambiguous whether the application of a diagnostic label is warranted, so the provision of a label simulates diagnostic expansion. Previous vignette research has instead typically employed clear or prototypical cases of mental illness. Second, this research examined the potential impacts of diagnostic labeling beyond effects on stigma. Based on studies of concept creep and concept breadth, it examined not only effects on positive or supportive responses but also effects on the person’s perceived suitability for receiving professional treatment and judgments of whether their problems were likely to persist and be under their control. Finally, this research employed vignettes describing conditions different from those employed in most previous vignette-based studies. Two experimental studies were conducted, the second aiming to replicate and extend the first.

## Study 1

Study 1 employed a vignette methodology to examine the effects of diagnostic labels on perceptions of people experiencing marginal levels of mental ill health. To assess the generality of any effects, the study included vignettes representing relatively mild symptoms of three mental disorders: Major Depressive Disorder (MDD), Bipolar Disorder (BD), and Generalized Anxiety Disorder (GAD). Participants were assigned to read all three vignettes either with the diagnostic label or without and completed four measures after each one. These measures assessed their empathic response to the person described in the vignette, their support for accommodations being offered to the person (e.g., extra leave from work, extensions on student assignments), their suitability for professional treatment, and the stability of their problems (i.e., how unlikely it would be for them to recover). We hypothesized that participants in the label condition would report more empathy towards the persons depicted on the vignettes, support more accommodations for them, perceive them as more suitable for professional treatment, and see their problems as more persistent than participants in the no-label condition. We made no predictions about differences between disorders but explored them as well as possible label × disorder interactions.

## Method

### Ethics statement

The study was approved by the University of Melbourne’s Human Research Ethics Committee (project 24553). Written formal consent was obtained. Participation was anonymous.

### Participants

Two hundred and sixty-two participants living in the U.S.A. were recruited from the online recruitment platform Prolific between 21/09/2022 and 23/09/2022. One participant was excluded for failing attention checks, leaving a final sample of 261. Participants ranged in age from 18 to 93 (mean = 39.08) and included 131 women, 121 men, six non-binary people, and three who did not say. The sample’s racial composition was primarily White (181; 69.4%), Black or African American (26; 10.0%), Asian or Pacific Islander (24; 9.2%), or Hispanic or Latino (19; 7.3%), with three participants (1.2%) reporting “other”.

### Materials

#### Vignettes

Participants completed a study in which they read three vignettes depicting a person experiencing symptoms that are marginal or near-threshold cases of different *DSM-5* disorders and made a series of ratings about each one before moving on to the next. The vignettes represented MDD, GAD, and BD were developed by Tse and Haslam [21], who wrote five variants of each vignette to capture degrees of severity from clearly failing to teach the diagnostic threshold to clearly exceeding it. As part of their pilot testing, Tse and Haslam had samples of 100 and 101 Prolific participants respond to each variant and rate it on the item “This person has a mental disorder” from 1 = *Strongly disagree* to 6 = *Strongly agree*. To select the vignettes for Study 1, we selected the variant of the MDD, BD, and GAD vignettes whose mean rating was closest to the scale midpoint of 3.5 to ensure they depicted optimally marginal or ambiguous cases (see S1 Appendix). Two versions of each vignette were written, the unlabeled version containing the description only and the labeled version preceding the same description with a sentence containing the diagnostic label (e.g., “This person has a diagnosis of Bipolar Disorder”).

After reading each vignette, participants completed four scales in randomized order. All were rated on 5-point scales, and with the exception of the Empathy scale, the response options were *strongly disagree*, *disagree*, *neither agree nor disagree*, *agree*, and *strongly agree*. Higher scores on each scale represent higher levels on each variable.

#### Empathy

State empathy was measured using an adjective rating task. Participants were asked to think about the person described in each vignette and rate how much they experienced eight emotions (sympathetic, touched, soft-hearted, compassionate, concerned, tender, moved, and sorrowful) found to load on a single empathy factor by Negd et al. [22]. The response options were *not at all*, *very little*, *somewhat*, *very much*, and *completely*. The internal consistency of the scale was high, with Cronbach’s α ranging from 0.93 to 0.94 across the three vignettes.

#### Accommodations

To assess participants’ support for offering accommodations, we developed a scale asking for levels of agreement with six forms of special allowance in work, academic, relationship, and family contexts. Example items are “This person’s employer should not hesitate to offer them extra time to complete work-related tasks”, “If this person is a university student and they missed a number of consecutive required classes, their instructors should turn a blind eye” and “This person’s family should not be angry if they need to spend Saturday afternoon resting rather than going to an important event”. Internal consistency was high, ranging from 0.84 to 0.86 across the vignettes.

#### Treatment suitability

A four-item scale was developed to assess perceived appropriateness for professional mental health treatment. Example items are “This person should seek professional treatment for their problems” and “This person’s problems could be easily managed without professional treatment” (reverse-scored). Internal consistency was marginal, ranging from 0.68 to 0.75.

#### Stability

To assess the perceived stability of the person described in the vignette’s problems, participants were asked to think about them and rate how strongly they agreed or disagreed with the four statements (e.g., “This person is likely to fully recover from their problems” [reverse-scored] and “This person’s problems are likely to continue throughout their life”). Internal consistency was marginal, ranging from 0.63 to 0.70 across the three vignettes.

### Procedure

After reading a short description of the study, participants were randomly assigned to the label or no-label condition and read the three labeled or unlabeled versions of the vignettes, respectively, in randomized order. All participants responded to four short questionnaire measures after each vignette and were then debriefed.

## Results

Repeated measures (mixed design) analyses of variance (ANOVAs) were conducted to test each of the hypotheses, and their results are summarized in Table 1. The main effects for labeling condition supported three of the four hypotheses. Participants reported higher empathy for the people depicted in the labeled than in the unlabeled vignettes (means = 3.43 vs 3.15), judged them more suitable for treatment (4.13 vs 3.81), and believed their problems were more likely to endure (3.11 vs 2.74). The weak trend for viewing the labeled vignettes as warranting more accommodations (3.44 vs 3.30) was not statistically significant.

**Table 1:** Summary of ANOVA findings, Study 1.

	Label effect		Disorder effect		Interaction effect	
	*F*(1,259)	*p*	*F*(1,518)	*p*	*F*(2,518)	*p*
Empathy	6.37	.012	57.53	< .001	10.92	< .001
Accommodations	2.15	.144	36.07	< .001	6.49	.002
Treatment suitability	18.86	< .001	6.27	.002	25.77	< .001
Stability	32.74	< .001	3.26	.038	29.56	< .001

Ratings on all four scales varied significantly between disorders. Post hoc Tukey HSD tests indicated that MDD elicited more empathy, was seen as more appropriate for receiving accommodations and professional treatment and was viewed as more persistent than GAD and (aside from treatment suitability) than BD. Significant interaction effects were also obtained for all scales, revealing that the effect of diagnostic labeling on empathy, accommodations, and stability was only significant for BD, and was only significant for BD and MDD for treatment suitability.

## Discussion

Study 1 found substantial support for its predictions. Participants reading about someone who was experiencing a marginal level of mental ill health that was not clearly diagnosable judged the person differently when a diagnostic label was affixed to them. Labeling led to judgments that are in most respects favorable but that might also be disadvantageous. The person elicited more empathy and was seen as more suitable for professional treatment but was also believed to have problems that would persist. This perception of persistence could be interpreted positively as evidence that the person’s difficulties are serious rather than trivial, but it could also contribute to prognostic pessimism, reflecting the concern that diagnostic labels can make problems seem part of the affected person’s identity.

The significant effects of labeling on empathy and perceived suitability for treatment have different implications. The former effect runs directly counter to fears that diagnostic labels promote stigma and suggests that it can generate compassion and sympathy instead. If the effect translates into real-world behavior, people might be rewarded interpersonally for disclosing professionally assigned diagnoses and incentivized to self-diagnose. The latter effect implies that a diagnostic label may ratify or legitimate relatively mild or marginal mental health problems and encourage people to seek professional help. Nevertheless, the lack of a significant effect on accommodations suggests that diagnostic labeling did not strongly legitimate the provision of allowances at home, work, or school.

Although most of the predicted labeling effects were statistically significant, they were all qualified by interaction effects, indicating that diagnostic labels had differential effects across the three disorders. Notably, labeling had no consistent effects on judgments of the GAD vignette and only one effect on the MDD vignettes, whereas it reliably had the hypothesized effect on judgments of BD. The reason for these differences is unclear but may reflect less familiarity with bipolar phenomena than with anxiety and depression. On this reading, the BD label gives psychiatric legitimacy to experiences that many people do not spontaneously see as disordered, resulting in increased empathy and perceived seriousness (persistence and need for treatment and accommodations). For more well-known conditions, labeling is not required for perceived legitimacy.

Study 1 had several limitations. First, two scales had relatively weak reliability. In particular, the Stability scale’s reliability was modest and follow-up analysis indicates that several items directly associated with the temporal persistence of the person’s problems were only weakly associated with other items whose content related to their capacity to control their problems. Although having control or agency over one’s problems should entail that they are less likely to persist, these elements of stability and personal controllability appear to be psychometrically distinct and should be assessed separately. Second, the repeated measures design, in which all participants judged three vignettes of the same (label or no-label) condition, might not be ideal for evaluating the labeling manipulation. The power of the manipulation might decline through repetition and its impact on the first vignette participants read might bleed into the second and third, making it impossible to assess the impact of labeling on each vignette separately. Finally, the study only examined judgments of three disorders, and especially in view of the label × disorder interaction effects, it is important to establish whether labeling effects replicate in another sample of disorders. A second study was therefore designed to improve measurement, simplify the experimental design, and replicate findings with a new set of vignettes.

## Study 2

Study 2 followed a very similar methodology to Study 1 but with adjustments to overcome limitations. The Treatment Suitability scale was lengthened to increase its reliability and the Stability scale was divided into separate Stability and Controllability scales, with items added to ensure adequate reliability for each new scale. We note that stability and controllability are typically distinguished within attributional research [23]. Study 1’s repeated measures design was replaced by one in which participants were rated a single vignette in a 2 (label/no-label) × 3 (disorder) design. To maintain statistical power and ensure large subsamples rated each vignette version, the sample size was raised substantially. The generality of effects was further tested by using vignettes representing marginal examples of three new disorders: post-traumatic stress disorder (PTSD), obsessive-compulsive disorder (OCD), and binge eating disorder (BED). Finally, a new measure of identity centrality was added to the study to examine whether the presence of diagnostic labels might lead people to infer that the person depicted in the vignette sees their problems as a key aspect of who they are, as noun categories have been claimed to do [18]. We hypothesized that participants in the label condition would report higher empathy, greater support for accommodations, greater perceived stability, lower perceived controllability, and higher perceived identity centrality than participants in the no-label condition.

## Method

### Participants

Seven hundred and six participants living in the U.S.A. were recruited from Prolific between 28/09/2023 and 29/09/2023. Twenty-two participants were excluded for failing to pass attention checks or finish the survey, resulting in a final sample of 684. Their mean age was 40.86 (range 18–80) and they included 335 women, 333 men, and 16 who were non-binary or preferred not to say. The sample’s racial composition was primarily White (475; 69.4%), Black or African American (85; 12.4%), Asian or Pacific Islander (55; 8.0%), or Hispanic or Latino (39; 5.7%).

### Design and procedure

Participants took part in a between-subjects experiment that was modeled on Study 1 but differed in three ways. First, they judged a single vignette (selected randomly from three alternatives) rather than three. Second, the vignettes referred to different *DSM-5* disorders. Third, some measures were added or revised in an effort to boost reliability. After being directed to a Qualtrics survey from the Prolific platform, they read a brief description of the study and provided consent to take part. Participants were randomly assigned to the label or no-label condition and then randomly assigned to read a vignette about a person with a marginal case of either PTSD, OCD, or BED (see S1 Appendix). As in Study 1, these vignettes were developed by Tse and Haslam [21] and pilot tested to be optimally marginal (i.e., mean rating near the center of the scale assessing agreement with the statement “This person has a mental disorder”). In the label condition, the vignette was preceded by “This person has been diagnosed with [diagnostic label]” and in the no-label condition it was not. The study was approved in advance by the University’s Institutional Review Board.

### Measures

Participants completed six scales in randomized order. The 8-item Empathy scale (Cronbach’s α = 0.92) and the 7-item Accommodations scale (α = 0.87) were identical to Study 1. Two items were added to the Treatment Suitability scale (now 6 items; α = 0.84) in a successful attempt to increase its psychometric reliability. Study 1’s Enduringness scale was split into separate Stability and Controllability scales with the same rationale, and new items were added to both. The 6-item Stability scale (α = 0.77) focused on the persistence of the person’s difficulties (e.g., “This person’s problems will likely persist”) whereas items in the 8-item Controllability scale (α = 0.75) focused on the person’s capacity to overcome their problems (e.g., “Recovery is out of this person’s control" [reverse-scored]). Finally, a 6-item Identity Centrality scale (α = 0.81) was adapted from a measure of racial identity centrality [24] to assess the perceived importance of the mental health problem to the person’s identity (e.g., “Overall, this person’s problems are an important reflection of who they are” and “In general, having these problems is an important part of this person’s self-image”). All items were rated on 5-point scales.

## Results

A summary of the two-way ANOVAs examining the six scales is presented in Table 2. Significant labeling effects, all in the predicted direction, were obtained for three scales. Participants reading vignettes that contained a diagnostic label judged that the person described deserved more accommodations (means 2.56 vs 2.44), was more suitable for professional treatment (4.05 vs 3.91) and had less control over their problems (3.01 vs 3.17) than those who judged otherwise identical unlabeled vignettes. There was a weak but consistent trend for the labeled vignettes to be judged as reflecting more stable or unchanging difficulties than the unlabeled vignettes (2.78 vs 2.71), but it did not reach statistical significance despite the large sample. Participants did not express any more empathy for the people depicted in labeled vignettes (3.30 vs 3.26) or infer that their problems were more central to who they were (3.10 vs 3.05).

**Table 2:** Summary of ANOVA findings, Study 2.

	Label effect		Disorder effect		Interaction effect	
	*F*(1,678)	*p*	*F*(2,678)	*p*	*F*(2,678)	*p*
Empathy	0.34	.558	18.16	< .001	0.19	.831
Accommodations	4.66	.031	76.86	< .001	1.94	.145
Treatment suitability	6.65	.010	2.94	.054	8.76	< .001
Stability	2.44	.119	50.60	< .001	0.17	.846
Controllability	12.63	< .001	68.12	< .001	3.73	.024
Identity centrality	0.88	.350	54.34	< .001	1.11	.329

Table 2 also presents several strong effects for the disorder described in the vignettes. Post hoc Tukey HSD tests indicated that participants rated the PTSD vignette particularly high for empathy and accommodations, rated the OCD vignette particularly high for stability, rated the BED vignette particularly high for controllability, and rated the OCD and BED vignettes particularly high for identity centrality. Two significant labeling by disorder interactions also emerged, both implicating BED. The presence of a diagnostic label for this disorder was especially potent in boosting perceived suitability for treatment and in reducing the perceived controllability of symptoms.

## Discussion

Study 2 substantially repeated Study 1 but with a simplified experimental design, some modified scales, a new measure of interest, and vignettes depicting a new set of (marginal) disorders. Its results, which support three of the six stated hypotheses, present a mixture of replications, non-replications, and new findings. The finding that the presence of a diagnostic label increases perceived suitability for professional treatment is a clear replication of Study 1, whereas the null effect for empathy is a clear non-replication. The significantly greater support for accommodations in the diagnostic label condition was not obtained in Study 1, although it found a nonsignificant trend in the same direction, suggesting that the accommodations effect is credible. Study 2’s significant effect of diagnostic labeling on controllability and its null effect on stability arguably clarify rather than failing to replicate the significant stability effect from Study 1. Study 1’s scale lacked reliability and appeared to combine stability- and controllability-related items, and the purified and somewhat more reliable Study 2 measures suggest that diagnostic labels reduce the perceived personal controllability of mental ill health more than they increase its perceived persistence. Finally, Study 2 obtained no support for the hypothesized effect of labels on identity centrality. Diagnostic labels did not lead participants to judge that the person’s problems were more personally defining.

As in Study 1, Study 2 supplied some evidence that the effects of labeling are moderated by disorder. The BED label was more impactful than MDD or GAD in leading participants to see the person in the marginal BED case as requiring professional treatment and as lacking control over their problems. Consistent with the interpretation offered in Study 1, this interaction may reflect the unfamiliarity of BED relative to PTSD and OCD. However, it might also reveal a tendency to normalize the BED vignette when no label is present. Only when disinhibited eating is legitimized as a disorder do participants infer that the person cannot control it and judge that it requires professional help rather than better self-control.

## General discussion

The two experimental studies offer considerable support for our predictions. Six of the ten hypothesis tests reached statistical significance, the four nonsignificant tests trended in the predicted direction, and all but one hypothesis was supported in at least one study. The prediction that labels promote the perception that the person is suitable for professional treatment was supported in both studies, and predictions that labels foster greater empathy, support for accommodations, and perceptions of stability and uncontrollability were each supported in one study. Only Study 2’s prediction that labels would lead people to believe that the person’s mental health problems are a more central part of their identity was not supported. The meaning of this null effect is ambiguous because the identity centrality scale contained items concerning centrality from both first- and third-person perspectives, referring to whether the person *saw* their problems as part of their self-image and whether those problems were central to who they were, respectively. Diagnostic labels might lead perceivers to see another person’s problems as central to who they are, but not lead the perceiver to infer that the *other person* sees their problems as self-defining. Clarifying this issue is a question for future research.

Our findings point to the potential mixed blessings of diagnostic labels. On the positive side, they suggest that the presence of a label can boost benevolent responses to the person who is experiencing mental ill-health, increasing empathy, support for flexible accommodations, and encouragement of help-seeking. If these effects translate into real-world behavior, they imply that diagnostic labels increase not only interpersonal support for people experiencing relatively mild or marginal mental ill-health but also support for institutional and professional intervention. These favorable impacts are a far cry from claims made in modified labeling theory [25] that labels increase stigma and communicate social rejection and devaluation. Destigmatization may have proceeded to the point that cultural beliefs about some forms of mental illness are now relatively positive.

On the more negative side of the ledger, however, our findings suggest that diagnostic labels also lead perceivers to believe that the labeled person’s problems are more likely to persist and that they have less control over them. If these findings are robust, they imply that labels may contribute to prognostic pessimism, often considered a dimension of stigmatizing attitudes [26], and to the view that people with mental ill health lack agency. If these labeling-related views are held by people experiencing mental ill-health, they may undermine expectations of their recovery and efforts to overcome their problems. These potential downsides of diagnostic labels are arguably especially concerning in the context of marginal cases, as these relatively non-severe experiences may be most likely to remit and to be responsive to self-help initiatives.

In that context, it is also important to acknowledge that the apparently positive implications of labelling may also have some downsides. Empathic responses to people experiencing relatively mild levels of mental ill-health are surely better than unempathetic ones, but they may reinforce diagnosis-based identities that have self-limiting aspects. Endorsement of special allowances can provide beneficial flexibility to people in distress, but they can also reinforce avoidance and adoption of a sick role. Diagnostic labels may promote professional help-seeking, but mental health services may become stretched if people at the less severe end of the spectrum seek treatment [27]. People with milder problems may also be less likely to benefit from professional treatment and may even tend to get worse [28]. Recognizing the possibility that concept creep might lead to overuse of mental health services in no way denies the reality that many populations lack adequate access to treatment, that there is significant unmet need for services, and that people in need should be encouraged to seek professional help. More research is required to evaluate these possible effects, but they suggest that it would be a mistake to view the increasingly favorable responses to diagnostic labels as invariably desirable.

Our findings have implications for research on concept creep and concept breadth. Historical studies have documented that mental health-related concepts have broadened over recent decades [9], have come to be used in less emotionally intense contexts [12], and have become more frequently accompanied by pathological language [13]. Cross-sectional studies also show that people with broad concepts of mental illness are more likely to self-diagnose, holding constant their levels of distress and impairment [14]. These findings all imply that people have become more likely to apply diagnostic labels to their own and others’ experiences, especially when these are at the milder end of the spectrum. Our findings suggest that when people do so they are increasingly likely to be helped and supported, but also increasingly likely to be seen as having enduring problems over which they have limited control. These positive outcomes may also encourage further concept creep by making diagnosis-based identities socially rewarding.

Our studies have several limitations. Like all vignette studies, their results may not generalize from online judgments to more ecologically realistic situations and real-life behaviors. Although they had relatively large samples, they may not have been powerful enough to detect subtle effects, potentially reducing the replicability of the empathy and accommodations findings across the two studies. Although they employed well-validated vignettes representing six *DSM-5* disorders, it is unclear whether the findings would generalize to other conditions or why they varied by disorder. Diagnostic labeling effects may be most powerful for disorders that are unfamiliar or readily normalized, and weak or non-existent for other disorders.

Determining the moderators of the labeling effects observed in our studies is an important question for future research. First, it should be asked whether more severe disorders or those with more stigmatizing labels, such as schizophrenia, tend to have less favorable labeling effects. Second, researchers might explore whether characteristics of participants or perceivers influence these effects. Younger and more politically progressive people tend to hold broader concepts of mental illness and are more liable to apply diagnostic labels to themselves [14], but whether labeling has differing implications for these groups is unclear. It is possible that they apply diagnostic labels more liberally but also draw fewer or less ambivalent inferences from them. The present studies’ random assignment to experimental conditions precluded investigation of the role of individual or demographic group differences on labeling effects, but they are promising avenues for future work. Equally, it is important to investigate whether labeling effects vary depending on the characteristics of targets–the people who are assigned diagnostic labels–as these effects are potentially more negative for members of marginalized groups. Whether the implications of labeling relatively mild experiences vary by gender and race, for example, is an urgent question for researchers.

The present research is the first to investigate the effects of diagnostic labels when they are applied to mild or marginal cases of mental ill-health. It illuminates the complex implications of labeling in the grey zone where the issue of whether or not a diagnosis is warranted is most salient. Studies of concept creep indicate that this grey zone has shifted towards less severe phenomena in recent years, and studies of concept breadth show that people differ in where they place it. Clarifying the positive and negative implications of applying diagnostic labels at the milder end of the mental health spectrum is a vital question for mental health researchers.

## Supporting information

S1 Appendix Appendices. (DOCX)
