# Supplementary material for: Effects of diagnostic labels on perceptions of marginal cases of mental ill-health
Source: PLOS Ment Health. 2024 Aug 28;1(3):e0000096. doi: 10.1371/journal.pmen.0000096 (PMC12798618; doi:10.1371/journal.pmen.0000096)
Supplement: S1 Appendix — (DOCX) [file pmen.0000096.s001.docx]

**Appendices**

**Study 1 vignettes (no label versions)**

**Major Depressive Disorder**: This person has been feeling very sad, self-critical, and upset every day for the past month. They have lost some weight and cannot fall asleep at night, so they feel tired all the time. They are also unable to concentrate and think clearly, which has impacted their work performance.

**Bipolar Disorder**: This person used to be very calm and quiet, but over the past two weeks, they have become much more talkative and easily irritated, which has caused a lot of conflicts with their friends. When asked about these changes, the person explained that they feel restless, that their thoughts are going faster than usual, and that they have trouble controlling their thoughts and behaviours.

**Generalized Anxiety Disorder**: This person is very anxious about many parts of their life. On and off throughout the day, they worry a lot about big things like getting fired, and sometimes about little things like forgetting to bring their keys. They find it very difficult to stop worrying. These anxieties often tense up their muscles, make them restless, and affect their concentration.

**Study 2 vignettes (no label versions)**

**Obsessive-Compulsive Disorder**: “Some days, this person thinks that they are contaminated in some way. The main way they can make the thoughts go away is to wash their hands with soap for at least 5 minutes. If they don’t wash their hands, they feel a little anxious. They have tried to stop their handwashing and can usually control it unless they are under stress. This problem causes some embarrassment with friends and at work.”

**Post-Traumatic Stress Disorder**: “This person witnessed a terrible car accident a month ago and since then they have been having nightmares about the accident every other night. The dream wakes them up in fear which keeps them awake and can sometimes last for the whole day. Although the accident and feeling are vivid in the dream, they fail to recall any specific details about the accident when asked. Because of this accident, they are easily startled by loud noises and they avoid the area where the accident had happened.”

**Binge-eating disorder**: “This person eats more than most people sometimes. Even when they don’t feel hungry, they eat a lot very quickly until they are uncomfortably full. Noticing this occasional pattern of over-eating for the past few months, they feel upset about it but also feel a little helpless about controlling how much they eat.”
